# G6PD promotes cell proliferation and dexamethasone resistance in multiple myeloma via increasing anti-oxidant production and activating Wnt/β-catenin pathway

**DOI:** 10.1186/s40164-022-00326-6

**Published:** 2022-10-21

**Authors:** Rui Li, Mengying Ke, Mingming Qi, Zhenru Han, Yuhao Cao, Zhendong Deng, Jinjun Qian, Ye Yang, Chunyan Gu

**Affiliations:** 1 Nanjing Hospital of Chinese Medicine Affiliated to Nanjing University of Chinese Medicine, Nanjing, China; 2 School of Medicine and Holistic Integrative Medicine, Nanjing University of Chinese Medicine, 138 Xianlin Road, Nanjing, China; 3 School of Integrated Chinese and Western Medicine, Nanjing University of Chinese Medicine, Nanjing, China

**Keywords:** Multiple myeloma, Glucose-6-phosphate dehydrogenase, Dehydroepiandrosterone, ROS, NADPH, Dexamethasone

## Abstract

**Background:**

Glucose-6-phosphate dehydrogenase (G6PD) as the rate-limiting enzyme in the pentose phosphate pathway (PPP) is well-established as an aberrantly expressed protein in numerous clinical diseases; however, its role in cancer, specifically in multiple myeloma (MM) remains elusive.

**Methods:**

In this study, serum metabolites in 70 normal people and 70 newly diagnosed MM patients were analyzed using untargeted metabolomics and the results were verified using ELISA. The survival analysis of multiple clinical datasets was performed to identify a potential target gene in MM. The oncogenic role of G6PD was investigated using lentivirus-based overexpression or knockdown of G6PD using RNAi or an inhibitor in vitro, and in a xenograft mouse model in vivo. The mechanisms of induced Dexamethasone (Dexa)-resistance of G6PD were further explored using the above established MM cell lines in vitro.

**Results:**

Based on the screening of potential genes, PPP was shown to be involved in the occurrence of MM, which was evidenced by the differential expression of serum metabolites of G6P and Dehydroepiandrosterone sulfate (DHEAS, the more stable sulfate ester form of an endogenously uncompetitive G6PD inhibitor known as DHEA). Elevated G6PD promoted MM cell proliferation. Mechanistically, high G6PD expression enhanced enzymatic generation of the antioxidant NADPH via the PPP and decreased the production of reactive oxygen species (ROS), thus inducing the proliferation and Dexa resistance in MM cells. Furthermore, canonical Wnt/β-catenin signaling also participated in regulating G6PD-induced drug resistance and cellular redox levels of ROS. Intriguingly, DHEA treatment could enhance the sensitivity of MM cells to Dexa primarily through augmenting cellular oxidative stress.

**Conclusions:**

Our data demonstrate that G6PD enhances the generation of the enzymatic anti-oxidant NADPH and decreases ROS generation, thereby promoting resistance to Dexa-induced apoptosis via the enzymatic PPP and non-enzymatic Wnt/β-catenin signaling pathway in MM. Targeting G6PD to harness cellular redox may serve as a promising novel strategy for the management of MM.

**Supplementary Information:**

The online version contains supplementary material available at 10.1186/s40164-022-00326-6.

## Background

Multiple myeloma (MM) is the second most common hematological malignancy worldwide. It was estimated that there were 34,920 new cases and 14,210 deaths in the United States in 2021 [1]. In 2017, there were 152,746 (140,564 to 172,662) incident cases of MM worldwide [2]. According to statistics collected in China, the incidence of MM in 2016 was 1.60 per 100,000 people [3, 4]. The last two decades have seen the development of cancer therapeutics from proteasome inhibitors (PIs, such as Bortezomib/BTZ) and immunomodulatory drugs (IMiDs) to antibody-based [5] or chimeric antigen receptor (CAR)-based [6, 7] targeting immunotherapies, all of which have achieved significant improvements in extending the survival of MM patients. However, most MM patients inevitably relapse, which is the most intractable problem regarding MM treatment [8]. Currently, the mechanisms driving the pathogenesis and progression of relapsed or refractory MM are poorly understood, and the additional hallmark of metabolic reprogramming in several tumors, particularly in MM, is poorly understood [9]. There is a growing interest in the pathways associated with reductive biosynthesis and redox homeostasis in cancer [10].

Glucose-6-phosphate dehydrogenase (G6PD) is an archetypical housekeeping gene encoding the first and rate-limiting enzyme (EC1.1.1.49) in the pentose phosphate pathway (PPP), which catalyzes glucose-6-phosphate (G6P) into 6-phosphogluconolactone [11]. PPP is the major metabolic pathway supplying the major source of reduced power nicotinamide adenine dinucleotide phosphate (NADPH) in the cytoplasm, and it serves as another route for glucose uptake and metabolism instead of glycolysis [12]. NADPH regenerates the essential antioxidant glutathione (GSH) that is vital for maintaining cellular redox homeostasis [11, 13]. The critical physiological and pathological roles of G6PD activity in maintaining redox homeostasis of healthy or diseased cells have been extensively discussed [14]. However, the mechanisms of G6PD activity on cell proliferation, survival, and chemo-resistance in MM have not been clearly defined.

Mounting evidence has demonstrated that G6PD expression is upregulated in several types of cancers and it promotes tumor progression [15]. Inhibition of G6PD has emerged as a potential therapeutic strategy for cancer treatment, such as the application of the small molecule G6PD inhibitor RRX-001 in colorectal cancer and hepatoma cells [16–18], and the endogenous G6PD inhibitor dehydroepiandrosterone (DHEA: 5-androsten-3beta-ol-17-one) in cervical and breast cancer cells [19, 20]. Interestingly, our previous untargeted UHPLC-MS metabolomic study identified changes in unique metabolites in the serum samples from MM patients and healthy individuals, particularly the reduction of dehydroepiandrosterone sulfate (DHEAS). DHEAS is the more stable sulfate ester form of DHEA in the blood, and both can be interconverted into each other via de-sulfation/sulfation via the action of hydroxysteroid sulfur transferase (HST) or steroid sulfatase (STS), respectively [21, 22]. As a metabolic precursor for the synthesis of steroid androgen and estrogen [23], DHEA possesses uncompetitive inhibition activity of G6PD, thus targeting the PPP [24, 25].

The present study was aimed to investigate the oncogenic role of G6PD in MM progression. We constructed G6PD knockdown (G6PD-KD) and overexpression (G6PD-OE) MM cell lines and established a MM cell line-derived xenograft (CDX) mouse model to explore the functions and potential mechanisms of G6PD in MM. The mechanistic studies demonstrated that G6PD was involved in the regulation of anti-oxidative activity and the Wnt/β-catenin signaling pathway. Our data provide novel insight into the action of G6PD as a promising therapeutic target for MM treatment, particularly in relapsed MM patients.

## Methods

### Untargeted metabolomics analysis

A total of 70 healthy individuals were recruited at the Affiliated Hospital of Nanjing University of Chinese Medicine, and 70 newly diagnosed MM patients were enrolled from the Shanghai Changzheng Hospital in 2018. The serum samples from all subjects were collected and stored in a digital-alarm-controlled freezer at − 80 °C until required for analysis. Sample preparation for the untargeted metabolomics analysis was performed as described in a previous study [26]. The Thermo Scientific^™^ Vanquish^™^ Horizon UHPLC system coupled to a Thermo Scientific^™^ Q Exactive hybrid quadrupole-Orbitrap mass spectrometer (Thermo Fisher Scientific, Inc., Waltham, MA, USA) operated in full scan mode was used for the untargeted analysis of serum samples. For the analyses carried out in the negative ESI mode, mobile phase A consisted of water containing 0.1% (vol/vol) formic acid (#F809712, Macklin, Biochemical Co., Ltd, Shanghai, China) and mobile phase B was acetonitrile (#1.00030.4008, Merck, Carrigtwohill, Ireland). The gradient profile was presented as previously described [27]. Data processing and statistical analysis was performed using SIMCA 14.1 software (Umetrics, Sweden) and MetaboAnalyst 5.0 (McGill University, Montreal, Quebec, Canada). The metabolites with a *p* value < 0.05 and a variable importance in the projection (VIP) of  > 1.0 were considered as significantly differentially expressed metabolites.

### Measurement of serum DHEAS levels

After thawing at 4 °C, the serum samples were used for measuring the concentrations of DHEAS by utilizing a human DHEAS ELISA kit (YFXEH00908, YIFEIXUE BIO TECH, Jiangsu, China). A total of 21 serum samples were selected randomly from both the healthy individuals and the MM group in duplicated wells for each assay. According to the manufacturer’s instructions, two identical treatments were performed. All the experimental operations were carried out by the same operator.

### Myeloma cell lines and cell culture

Human MM cell lines ARP1 and H929 were kind gifts from Prof. Zhiqiang Liu (Department of Physiology and Pathophysiology, School of Basic Medical Science, Tianjin Medical University). MM.1S and MM.1R cells were purchased from ATCC (CRL-2974 and CRL-2975, respectively). Mouse 5TMM3VT cells were donated by Dr. Wen Zhou (Xiangya School of Medicine, Central South University, Key Laboratory of Carcinogenesis and Cancer Invasion, Ministry of Education; Key Laboratory of Carcinogenesis, National Health and Family Planning Commission, Changsha, China). The cells were maintained at 37 °C with 5% CO_2_ in RPMI 1640 media (#05-065-1A, Biological Industries, Beit Haemek, Israel) supplemented with 10% heat-inactivated fetal bovine serum (FBS; #04-002-1A, Biological Industries, Israel) and 1% penicillin/streptomycin.

### Cell proliferation

MM cell proliferation was assessed using a colorimetric MTT assay. The cells were seeded in a 96-well plate at a density of 5×10^3^ cells per well containing 180 μL complete medium. After 48 h, 20 μL MTT (5 mg/mL) was added to each well. After culturing for 4 h, the supernatant was removed by centrifugation (4000 rpm, 15 min, room temperature) and dimethyl sulfoxide was added to each well. Then a microplate reader (Thermo Fisher Scientific, Inc., USA) was used to measure the absorbance at 570 nm. Growth curves showed absolute cell numbers counted using a hemocytometer and trypan blue staining.

### Cell cycle analysis

The cells were washed twice with ice-cold PBS and fixed with 75% ice ethanol overnight. The following day, after washing the cells with PBS, RNase A (200 μg/mL) was added to treat the cells on ice for 1 h. Then 50 μg/mL PI (#25535-16-4, Sigma-Aldrich, Merck KGaA, Darmstadt, Germany) was added, and cells were incubated for 5 min at room temperature, after which flow cytometry (Guava Technologies, Hayward, CA, USA) analysis was performed. The flow cytometry data were analyzed using ModFit LT version 5.0 (Verity Software House, Topsham, ME).

### Apoptosis analysis

MM cells were seeded in a 6-well plate with 1.5×10^6^ cells per well. The administration group was treated with Dexa (#D4902, Sigma-Aldrich, Merck KGaA, Darmstadt, Germany) or RRX-001 (#925206-65-1, Shanghai yuanye Bio-Technology, Shanghai, China), and cells were subsequently incubated at 37 °C in a humidified incubator supplied with 5% CO_2_ for 48 h. The cells were collected in a centrifuge tube, centrifuged at 1200 rpm for 5 min at room temperature, after which the supernatant was discarded, the pellet was washed twice with pre-cooled PBS, and following centrifugation as above, the supernatant was discarded again. Subsequently, cell pellets were resuspended in 100 μL 1 × Binding buffer, to which 5 μL Annexin V-APC (#640920, Biolegend, Beijing, China) and 5 μL PI (50 μg/mL, #25535-16-4, Solarbio Life Science, Beijing, China) were added, the cells were incubated at room temperature for 5 min, and 400 μL 1 × Binding buffer was finally added. The samples were loaded on Guava easyCyte6-2L flow cytometer (Millipore Corp, Bedford, MA,USA) for detection and analyzed using GuavaSoft3.1.1 (Merck KGaA, Darmstadt, Germany).

### Animal experiments

The experimental mice were housed in the SPF laboratory animal center of Nanjing University of Chinese Medicine with 15–25 °C ambient temperature and freely access to food and water. ARP1 WT and G6PD-OE cells (1 × 10^6^) were subcutaneously injected into the abdominal region of 6 to 8 week-old NOD-SCID mice (n = 6 per group) from Beijing Vital River Laboratory Animal Technology, Co., Ltd (Beijing, China). The tumor volumes were measured using calipers at the indicated time points. When the tumor diameters reached 20 mm, photographs were recorded after anesthesia with an intraperitoneal injection of 1% sodium pentobarbital (50 μL/10 g) and the mice were sacrificed by dislocation of cervical vertebra. Tumor volume (mm^3^) was calculated as: (length×width^2^)/2. The 8 week-old male/female C57BL/KaLwRij mice (n = 10 per group) were injected intravenously with 5TMM3VT (1×10^6^ cells per mouse). Treatment with RRX-001 was initiated from Day 2 (twice a week) and continued until the mice had died/were sacrificed. All animal procedures were conducted in accordance with government-published recommendations for the Care and Use of Laboratory Animals and approved by the Institutional Ethics Review Boards of Nanjing University of Chinese Medicine (No. ACU170501).

### ROS analysis

MM cells were cultured with different concentrations of DHEA (#D806984, Macklin Biochemical Co., Shanghai, China), DHEAS (#HY-B0765, MedChemExpress, New Jersey, USA), or Dexa at 37 °C in a humidified incubator supplied with 5% CO_2_ for 24 h. DCFH-DA (S0033, Beyotime Institute of Biotechnology, Shanghai, China) was diluted in serum free medium (dilution ratio: 1:1000). DCFH-DA was added to 1 × 10^6^ cells/mL at 1 µM concentration. The cell mixture was incubated at 37 °C for 20 min (mixed every 5 min) and washed three times with 1 × PBS. The fluorescence intensity of ROS production was measured at an excitation/emission wavelength of 488/525 nm in Guava easyCyte6-2L flow cytometer and analyzed using GuavaSoft3.1.1 and FlowJo_V10 software (Becton, Dickinson & Company, Franklin Lakes, NJ, USA).

### Western blot analysis

MM cells were harvested, washed, and lysed using RIPA lysis buffer (#FMS-WB035, Fcmacs Biotech Co., Ltd., Nanjing, China). Protein concentration were detected by the BCA protein assay kit (#P0011, Beyotime Biotechnology, Shanghai, China). Total protein samples (20–40 μg) were heated in SDS/β-mercaptoethanol buffer and loaded on 10–15% SDS-gels. The proteins were resolved using SDS-PAGE, and then transferred to a PVDF membrane. The membrane was blocked with 5% non-fat milk at room temperature for 2 h and incubated with primary antibodies against G6PD (1:1,000 dilution, #ab210702, Abcam, Shanghai, China), β-catenin (1:1 000 dilution, #51,067-2-AP, Proteintech Group, Wuhan, China) and β-actin (1:1000 dilution, #4970S, Cell Signaling Technology, Mass, USA) overnight at 4 °C. Blots were incubated with secondary antibodies using horseradish peroxidase-conjugated rabbit anti-mouse (1:10,000 dilution, #S0002, Affinity, OH, USA) or goat anti-rabbit IgG (1:10,000 dilution, #FMS-Rb01, Fcmacs, Nanjing, China) at room temperature for 1 h. Finally, blots were developed using a chemiluminescence ECL kit (#180-5001, Tanon, Shanghai, China).

### NADP^+^/NADPH assay

A NADP^+^/NADPH Assay Kit with WST-8 (#S0179) was purchased from Beyotime Biotechnology (Shanghai, China). The experiment was conducted according to the manufacturer’s instructions.

### Statistical analysis

All the experiments were repeated three times. Data are presented as the mean ± SD. Differences between two independent experimental groups were assessed using an unpaired Student’s t-test, while differences between multiple groups were compared using a one-way ANOVA method. The threshold p-values were set at *p* < 0.05 ( ∗ ), *p* < 0.01 (∗  ∗) and *p* < 0.001 (∗  ∗  ∗). All data were analyzed using GraphPad Prism 8.0 software (GraphPad Software Inc., La Jolla, CA, USA).

## Results

### Metabolomics profiling identifies a significant decrease of DHEAS in MM patients

**Fig. 1:**
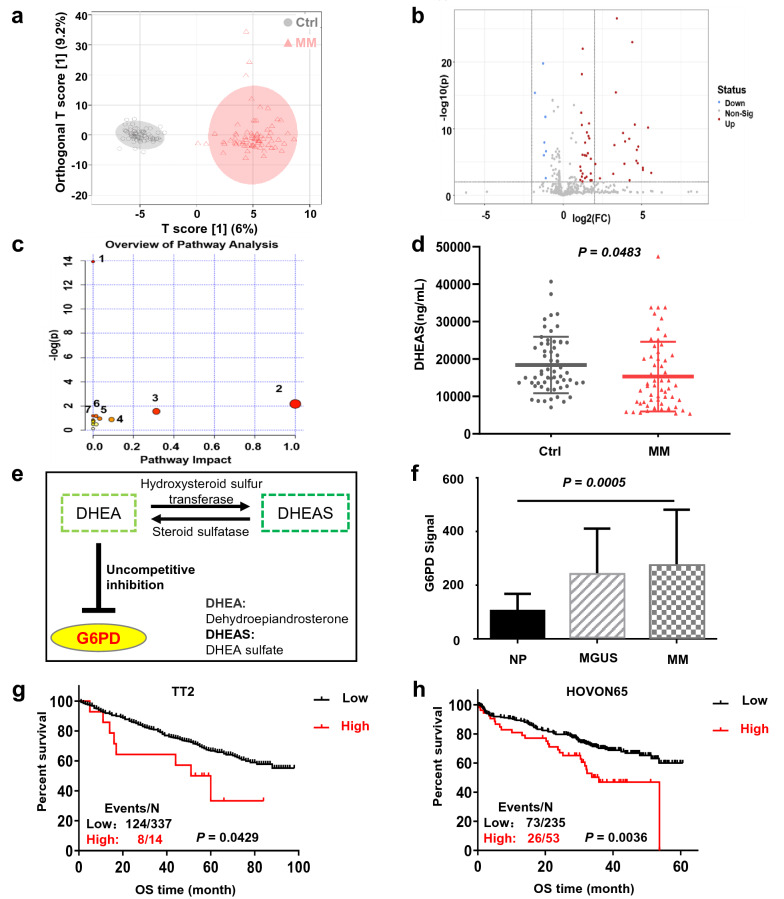
Metabolomics and GEO analyses indicate high G6PD expression is associated with poor prognosis in MM. **a** Score plot of OPLS-DA based on data from ESI^−^ mode in healthy people and newly diagnosed MM patients. **b** A volcano plot showed that there were 53 differentially expressed substances, including 46 upregulated and 7 downregulated substances in the serum of the MM group compared with the Ctrl group. **c** KEGG pathway enrichment analysis revealed the perturbed pathways. 1. Biosynthesis of unsaturated fatty acids. 2. Linoleic acid metabolism. 3. Arachidonic acid metabolism. 4. Pentose phosphate pathway. 5. Citrate cycle (TCA cycle). 6. Fatty acid biosynthesis. 7. Butanoate metabolism. **d** ELISA analysis showed serum DHEAS levels in healthy people and newly diagnosed MM patients. **e** Diagrammatic sketch of DHEA and its transformation to the more stable sulfate ester DHEAS by HST. **f** mRNA levels of G6PD were significantly increased in MM samples based on the one-way ANOVA. **g**, **h** Kaplan–Meier analysis showing the association between G6PD expression with OS in TT2 (**g**) and HOVON65 (**h**) cohorts based on a log-rank test

Untargeted metabolomics analyses were performed on the serum samples from 70 healthy individuals (Ctrl group) and 70 newly diagnosed MM patients (MM group, clinical characteristics shown in Additional file 1: Table S1). The resulting metabolic profiles were clearly segregated between Ctrl and MM groups based on Orthogonal Projections to Latent Structures Discriminant Analysis (OPLS-DA) (Fig. 1a). G6P and DHEAS were considered as significantly differentially expressed metabolites (Fig. 1b) with *p* values < 0.05 and VIP values of > 1.0 (Table 1). Kyoto encyclopedia of genes and genomes (KEGG) pathway enrichment analysis revealed that PPP was the representative perturbed pathway in MM with an impact value of 0.09162 (−log(p) = 0.88079) (Fig. 1c). Since G6P is competitively utilized by both of the Glycolysis pathway and PPP, it may not directly reflect PPP perturbation [28]. We measured the absolute serum DHEAS concentrations using triplicate repeats of ELISA, showing that there were statistically lower DHEAS levels in the MM group compared with the Ctrl group (n = 59, *p* = 0.0483, Student's unpaired two-tailed t-test) (Fig. 1d). Additionally, DHEAS could be interconverted freely into the endogenous uncompetitive inhibitor of G6PD, DHEA in the blood [21, 22, 24, 25] (Fig. 1e). Therefore, both untargeted metabolomics and quantitative ELISA data supported the further investigation of the relationship between the activity/expression of the core PPP enzyme G6PD with relation to the prognosis of MM.

**Table 1:** Top 10 differential metabolites between the serums of Ctrl group and MM group

Name	FDR	*p*.Value	VIP	Fold change	KEGG
Glucose-6-phosphate	4.20E−20	1.59E−21	1.0519	69.1344	C00092
5-OxoETE	1.78E−18	8.37E−20	1.8185	48.5672	C14732
Gamma-linolenic acid	1.04E−21	2.32E−23	1.3615	38.1415	C06426
9-Hydroxy-10,12-octadecadienoic acid	1.01E−21	1.46E−23	1.4092	37.4001	C14767
Eicosapentaenoic acid	9.78E−22	1.04E−23	1.7995	31.9019	C06428
Docosahexaenoic acid	5.89E−22	2.67E−24	1.9917	27.7889	C06429
Prostaglandin E1	1.63E−21	4.34E−23	1.9103	16.2286	C04741
5-HETE	3.74E−21	1.08E−22	2.6457	12.8818	C04805
Dehydroisoandrosterone sulfate	6.14E−07	9.69E−08	1.2119	12.0211	C04555
9-Hydroxy-10,12-octadecadienoic acid	1.55E−18	6.91E−20	1.7806	10.8084	C14767

### High expression of G6PD is associated with a poor prognosis in patients with MM

Based on publicly available datasets from NCBI Gene Expression Omnibus (GSE2658 and GSE5900), we found that G6PD mRNA expression was significantly increased during MM progression from normal bone marrow plasma cells (NP, n = 22), and in “premalignant” individuals with monoclonal gammopathy of undetermined significance (MGUS, n = 44) to malignant plasma cells of newly diagnosed MM (MM, n = 351) (Fig. 1f). This result was consistent with a recent report on 12 MM patients [29]. Furthermore, the Kaplan–Meier analysis indicated a statistically significant inverse correlation between G6PD levels and overall survival (OS) in MM patients from the clinical datasets of both the University of Arkansas Total Therapy 2 (TT2) and the Dutch-Belgian cooperative trial group for hematology oncology group-65 (HOVON65) cohorts (Fig. 1g, h). The above results suggested that upregulated G6PD expression was correlated with a worse clinical outcome in patients with MM.

### Elevated G6PD promotes cellular proliferation in MM cells and in a xenograft mouse model

**Fig. 2:**
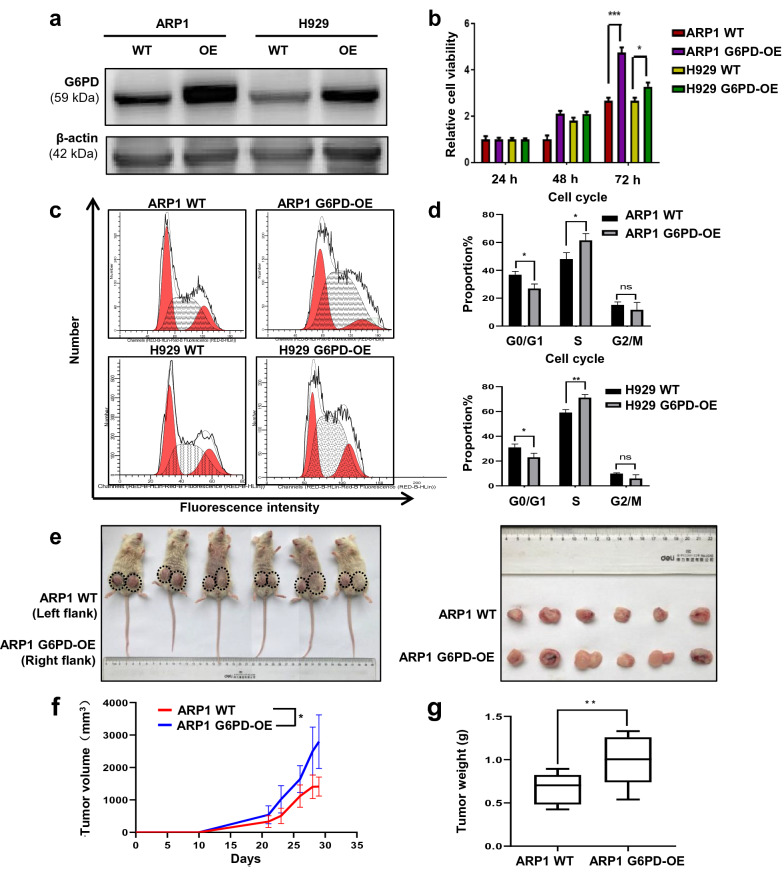
Elevated G6PD expression promotes cellular proliferation in MM cells and in the xenograft mouse model. **a** WB analysis confirmed that G6PD-OE MM cells successfully expressed upregulated levels of G6PD. **b** MTT assays were used to measure cell viability in WT and G6PD-OE MM cells. **c, d** Cell cycle distribution in WT and G6PD-OE cells was determined using flow cytometry (**c**) and the results are presented as histograms (**d**). **e–g** Increased G6PD expression significantly increased tumor growth (**e**, **f**) and tumor weight (**g**) in the xenograft mouse model. The difference between groups was calculated using a Student’s t-test. *p < 0.05, **p < 0.01 and ***p < 0.001 were considered statistically significant

To identify the functions of G6PD in MM cells, we constructed two G6PD-OE cell lines using ARP1 and H929 MM cells with a lentivirus expression system. WB analysis confirmed that the G6PD-OE MM cells were constructed successfully (Fig. 2a). MTT assays (Fig. 2b) and growth curves (Additional file 2: Fig. S1a, b) demonstrated notably enhanced cellular viability in the G6PD-OE MM cells relative to the wildtype (WT) MM cells (*p* < 0.05). Consistently, flow cytometry analysis showed that overexpression of G6PD induced cell cycle arrest at the G0/G1 phase while the proportion of cells in the S phase was increased in both ARP1 and H929 cells (Fig. 2c, d), suggesting that G6PD facilitated MM cell proliferation. Subsequently, we established an MM cell line-derived xenograft (CDX) mouse model to investigate the role of G6PD in vivo. A total of 2 weeks after MM cell injection, it was evident that the tumors in the G6PD-OE group developed faster than in the WT group (Fig. 2e, f). Accordingly, the tumor weight was significantly increased in the G6PD-OE group compared with the WT group (Fig. 2g).

### Deceased G6PD expression blunts MM cell viability and improves the survival of MM mice

**Fig. 3:**
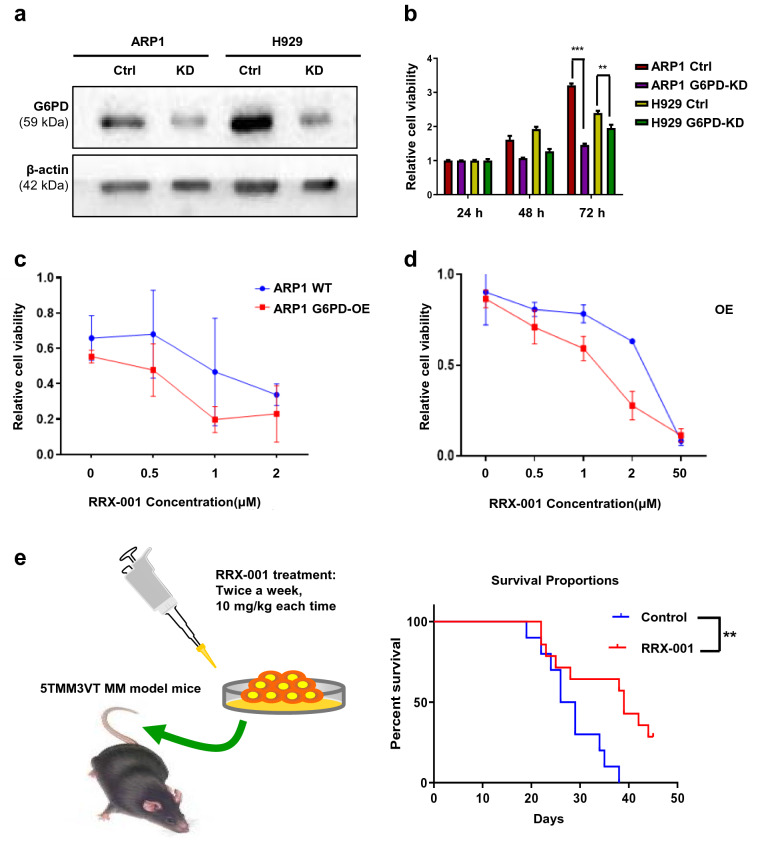
Knockdown of G6PD suppresses MM cell viability and improves the survival of MM mice. **a** WB analysis confirmed that G6PD-KD MM cells expressed significantly lower levels of G6PD. **b** MTT assays showed that decreased G6PD decreased MM cell viability. **c**, **d** The effect of RRX-001 treatment on cell viability in ARP1 (**c**) and H929 (**d**) WT and G6PD-OE cells. **e** RRX-001 treatment improved the survival of MM-prone C57BL/KaLwRij mice (n = 10). The differences between groups were calculated using a Student’s t-test. **p < 0.01 and ***p < 0.001 were considered statistically significant

To further confirm the role of G6PD in cellular proliferation, we utilized RNAi technology to knock down G6PD expression in the MM cell lines, using transiently expressed shRNA-G6PD induced by doxycycline (DOX). As shown in Fig. 3a, G6PD-KD MM cells were constructed successfully. MTT analysis (Fig. 3b) and growth curves (Additional file 2: Fig. S1c, d) showed that the cell viability was significantly decreased in the G6PD-KD cells compared with the control cells. In addition, we took advantage of RRX-001 (1-bromoacety l-3,3-dinitroazetidine) to suppress G6PD activity. RRX-001 is a G6PD inhibitor and multifunctional anticancer drug in phase III clinical trials [16, 18]. As shown in Fig. 3c, d, overexpression of G6PD increased the sensitivity of MM cells to RRX-001 compared with the WT cells (IC_50_/μΜ of RRX-001 in ARP1 WT: 1.75 vs OE: 0.997; H929 WT: 2.50 vs OE: 1.38). Next, we used the 5TMM3VT MM mouse model to test the protective effects of RRX-001 on the survival of MM mice in vivo (Fig. 3e). The conditions of the control mice rapidly deteriorated from Day 20 and all mice in the control group had died by Day 38. In comparison, RRX-001 treatment led to prolonged survival up to ~ 50 days in the MM mice (*p* = 0.0088).

### Increased G6PD expression enhances MM cell resistance to Dexa, but not BTZ.

**Fig. 4:**
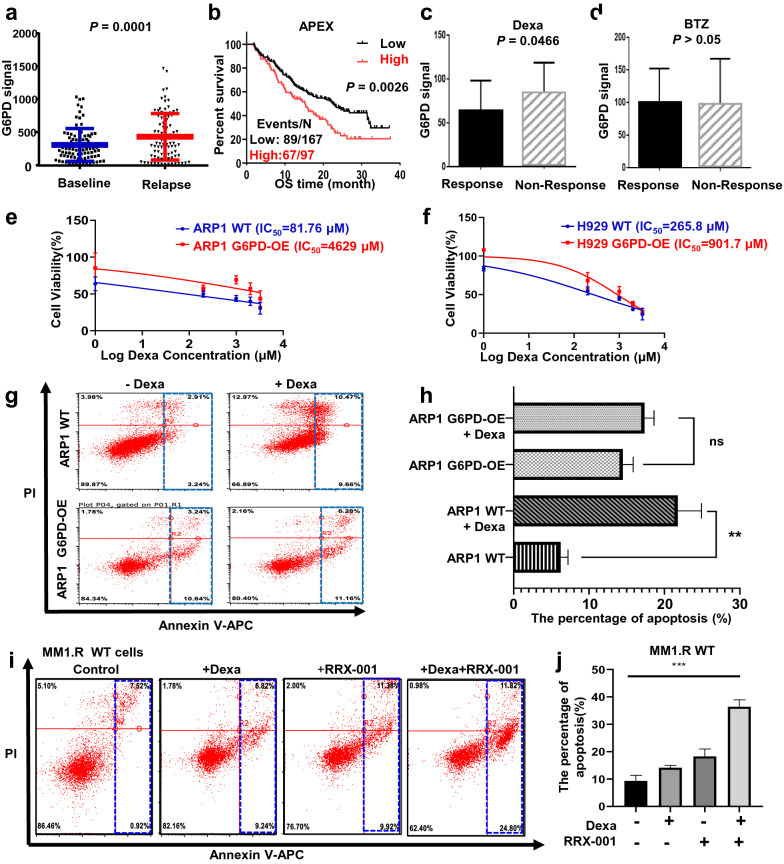
Increased G6PD expression enhances MM cell resistance to Dexa, but not BTZ. **a** The mRNA expression levels of G6PD were markedly increased in relapsed MM patients compared with baseline. Differences were compared using an unpaired t-test. **b** G6PD expression was associated with patient survival in the APEX clinical trial based on a log-rank test. **c** G6PD expression was higher in patients who did not respond to Dexa treatment compared with patients who did respond to Dexa treatment. **d** No significant difference in G6PD expression was observed between patients who did and did not respond to BTZ. **e**, **f** Overexpression of G6PD enhanced Dexa resistance in ARP1 (**e**) and H929 (**f**) G6PD-OE cells compared with the respective WT cells. **g**, **h** Flow cytometry analyses confirmed that overexpression of G6PD attenuated Dexa-induced apoptosis in G6PD-OE cells relative to WT cells. **i**, **j** Flow cytometry analyses indicated that MM1.R cells resistant to Dexa exhibited an increased degree of apoptosis upon RRX-001 treatment. ***p < 0.001 was considered statistically significant

To further explore the association between high expression of G6PD and the progression of MM, we examined G6PD expression in the recurrent MM patients from APEX (Assessment of Proteasome Inhibition for Extending Remissions) cohort. The Affymetrix U133 Plug 2.0 gene chip data indicated that the levels of the G6PD signal in the relapsed MM patients were significantly higher than the baseline, and G6PD expression was positively correlated with a worse outcome compared with the healthy individuals (Fig. 4a, b). Both BTZ and Dexa are first-line drugs. G6PD expression in the MM patients not responding to Dexa treatment was significantly higher than in patients who responded to Dexa treatment (*p* < 0.05) (Fig. 4c). In contrast, there was no difference in expression of G6PD in patients treated with BTZ based on response (Fig. 4d). This suggests that G6PD may be involved in the specific response to Dexa in MM patients.

In agreement with the above findings, MTT assays confirmed that G6PD-OE cells exhibited increased survival with a 4-5-fold IC_50_ higher dose of Dexa than the WT ARP1 (OE/WT: 4629/81.76) and H929 (OE/WT: 901.7/265.8) cells, respectively (Fig. 4e, f). The flow cytometry analysis demonstrated that Dexa treatment resulted in a significant increase in the proportion of apoptotic cells in the Dexa-treated WT cells compared with the untreated cells (*p* = 0.0335); however, Dexa did not trigger a similar level of apoptosis in the G6PD-OE cells (Fig. 4g, h). Additionally, the combination treatment of Dexa and RRX-001 on Dexa-resistant MM1.R cells evidently increased the proportion of apoptotic cells compared with Dexa or RRX-001 treatment alone (Fig. 4i, j).

### G6PD-mediated Dexa resistance in MM cells is related to cellular redox levels of NADPH and ROS

**Fig. 5:**
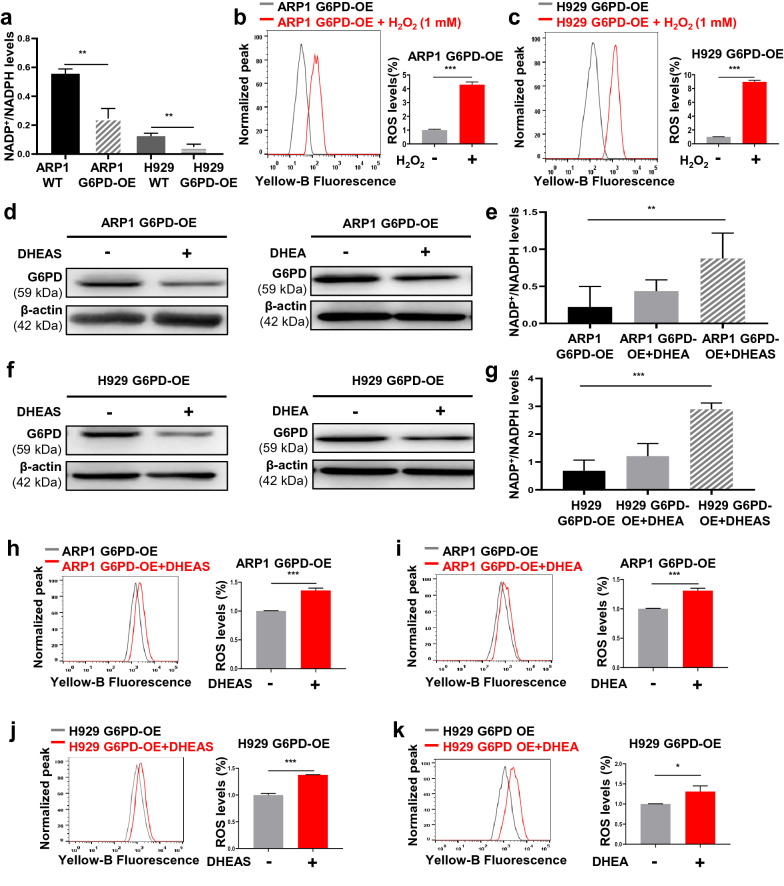
G6PD-mediated Dexa resistance in MM is related to cellular redox levels of NADPH and ROS. **a** Histogram depicting the relative NADP^+^/NADPH ratio in WT and G6PD-OE MM cells. **b**, **c** ROS content was increased by adding H_2_O_2_ (1 mM) in ARP1 (**b**), and H929 (**c**) G6PD-OE cells.** d** WB analysis showed that the expression of G6PD was reduced in ARP1 G6PD-OE cells treated with DHEAS or DHEA. **e** Histogram showing the relative NADP^+^/NADPH ratio in ARP1 G6PD-OE cells treated with DHEAS or DHEA.** f** WB analysis showed that the expression of G6PD was reduced whereas H929 G6PD-OE cells were treated with DHEAS or DHEA. **g** Histogram depicting the relative NADP^+^/NADPH ratio in H929 G6PD-OE cells treated with DHEAS or DHEA. **h**–**k** ROS content was increased following treatment of DHEAS or DHEA in ARP1 (**h**, **i**) and H929 (**j**, **k**) G6PD-OE cells. *p < 0.05, **p < 0.01 and ***p < 0.001 were considered statistically significant

Considering the central role of G6PD in the PPP for producing ribose and reducing equivalent NADPH, we measured NADPH content in MM cells. The NADP^+^/NADPH ratio was significantly lower in the G6PD-OE cells than in the WT cells, indicating increased NADPH content was generated upon G6PD overexpression (Fig. 5a). Next, we induced the production of ROS by adding 1 mM H_2_O_2_ into G6PD-OE cells to explore the association between anti-oxidative NADPH and ROS. The dihydroethidium (DHE) fluorescent probe exhibits blue fluorescence in the cytosol until it is oxidized following its intercalation into DNA, whereupon it emits bright red fluorescence. Supplementation of extra H_2_O_2_ significantly induced ROS generation by 4-fold in ARP1 G6PD-OE cells and by eightfold in H929 G6PD-OE cells compared with the respective control cells (Fig. 5b, c). Then we treated MM cells with 10 μM DHEA or 15 μM DHEAS for 24 h, respectively. WB analysis showed that DHEA or DHEAS significantly inhibited G6PD expression at the protein level (Fig. 5d, f). Higher NADP^+^/NADPH ratios were observed in G6PD-OE cells treated with DHEA or DHEAS compared with untreated cells (Fig. 5e, g), supporting the critical role of G6PD in producing NADPH. We further found that DHEA or DHEAS notably triggered increased ROS generation by ~ 1.5-fold in ARP1 G6PD-OE cells compared with the untreated cells (Fig. 5h, i). A similar result was observed in H929 G6PD-OE cells (Fig. 5j, k), supporting the notion that high levels of G6PD result in relatively low levels of oxidative stress in MM cells resistant to Dexa.

### The Wnt/β-catenin signaling pathway is involved in G6PD-induced Dexa resistance in MM

**Fig. 6:**
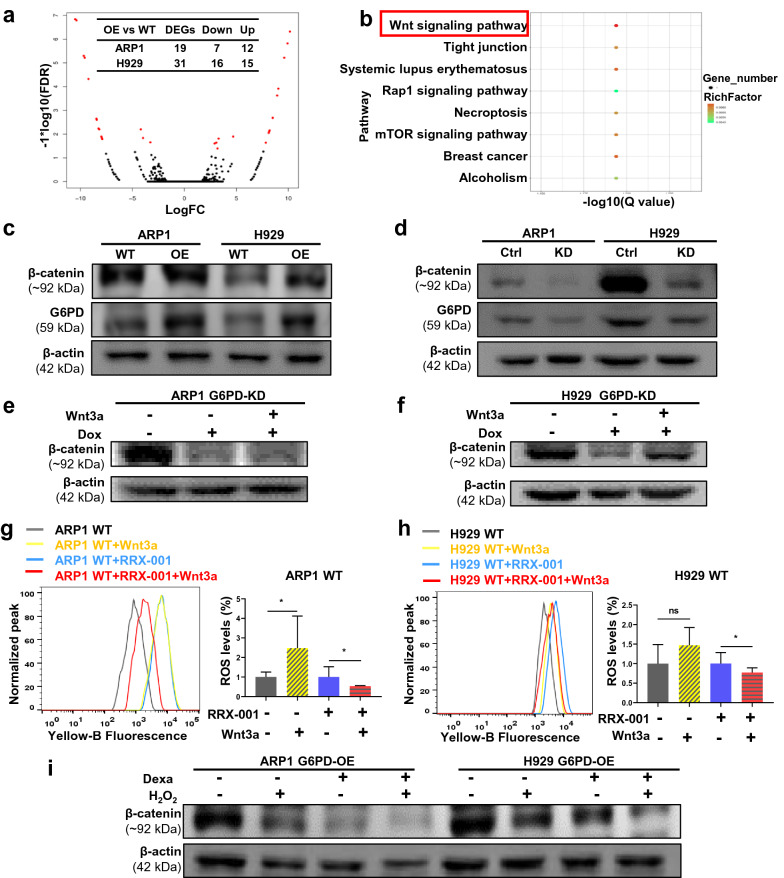
The Wnt/β-catenin signaling pathway is involved in G6PD-induced Dexa resistance in MM. **a** Volcano plot showing the differentially expressed genes (DEGs) identified by RNA-seq between the WT and G6PD-OE MM cells. **b** KEGG pathway analysis indicated that the DEGs were primarily enriched in the Wnt/β-catenin signaling pathway, the top enriched KEGG pathway. **c**, **d** WB analysis showed that the expression of G6PD was positively correlated with β-catenin in G6PD-OE (**c**) or G6PD-KD (**d**) MM cells. **e–f** WB analysis confirmed that Wnt3a rescued the decrease in β-catenin expression in ARP1 (**e**) and H929 (**f**) G6PD-KD cells. **g**, **h** Supplementation of Wnt3a decreased the ROS levels in ARP1 (**g**) and H929 (**h**) cells treated with RRX-001. **i** WB analysis of β-catenin levels in G6PD-OE cells treated with H_2_O_2_ and Dexa individually or combined. *p < 0.05 was considered statistically significant

Transcriptomic RNA sequencing (RNA-seq) technology was used to screen the downstream genes of G6PD in ARP1 and H929 WT and G6PD-OE cells. We found 19 differentially expressed genes (DEGs, 12 upregulated and 7 downregulated) in ARP1 cells and 31 DEGs (16 upregulated and 15 downregulated) in H929 cells (Fig. 6a). The Gene Ontology (GO) and KEGG enrichment analyses identified 8 signaling pathways as the top enriched pathways including the Wnt and mTOR (mammalian target of rapamycin) signaling pathways (Fig. 6b). We detected the expression of the components of the enriched pathways by WB analysis and found notably altered AKT protein expression (a member of the mTOR signaling pathway) in H929 cells but not in ARP1 cells (Additional file 2: Fig. S2a, b). Therefore, we focused on the Wnt/β-catenin signaling pathway to determine whether it was dysregulated by alteration of G6PD levels. As a core component of the Wnt/β-catenin pathway, β-catenin expression was increased in G6PD-OE cells and decreased in G6PD-KD cells (Fig. 6c, d). Furthermore, suppression of β-catenin in G6PD-KD cells was increased following administration of additional Wnt3a to the culture medium to activate the canonical Wnt pathway (Fig. 6e, f). To further validate the link between β-catenin and G6PD-mediated anti-ROS generation in Dexa-resistant cells, we examined the changes in ROS levels by adding Wnt3a to MM cells with or without RRX-001 treatment. The results showed that Wnt3a treatment effectively increased the ROS levels, whereas the elevation of ROS levels was significantly decreased in both cell lines when treated with RRX-001 (Fig. 6g, h). The critical contribution of the Wnt/β-catenin pathway to G6PD-mediated anti-ROS generation may be achieved in a G6PD-dependent manner in MM (Additional file 2: Fig. S3). Importantly, the upregulated expression of β-catenin in G6PD-OE cells was significantly decreased by Dexa and H_2_O_2_ individually and when combined (Fig. 6i & Additional file 3: Fig. S4). Collectively, the Wnt/β-catenin signaling pathway was shown to be involved in G6PD-induced Dexa resistance in MM cells.

### DHEA relieves G6PD-mediated Dexa resistance in MM cells

**Fig. 7:**
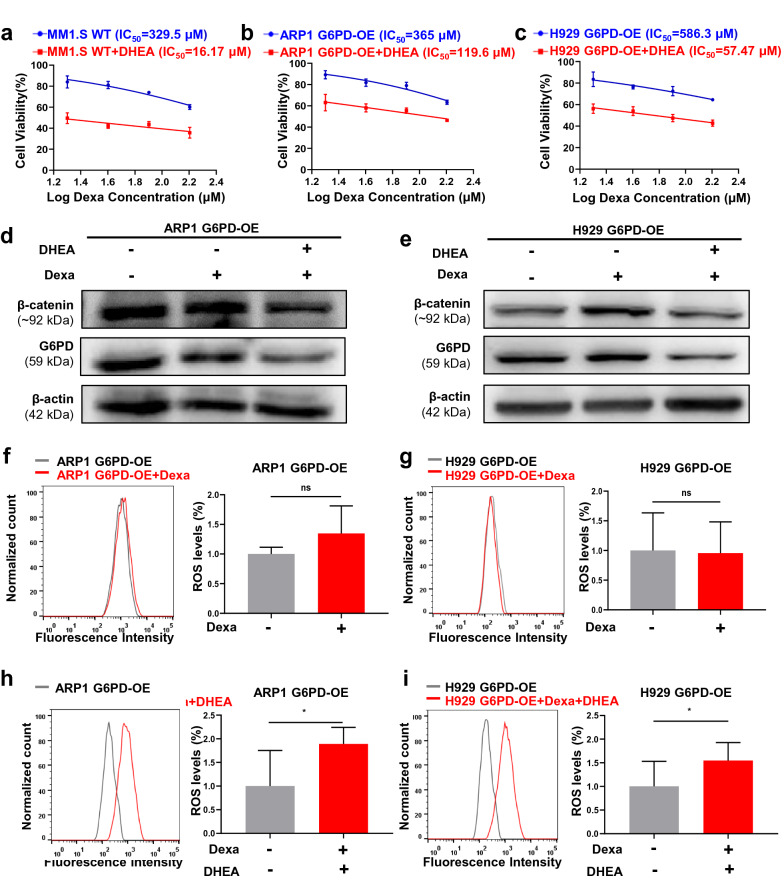
DHEA relieves G6PD-mediated Dexa resistance in MM cells. **a**–**c** The sensitivity to Dexa was assessed in MM1.S WT (**a**), ARP1 (**b**) and H929 (**c**) G6PD-OE MM cells treated with DHEA for 48 h. **d-e** WB analysis showed the expression levels of β-catenin and G6PD in ARP1 (**d**) and H929 (**e**) G6PD-OE cells treated with DHEA and Dexa individually or combined. **f**, **g** Intracellular oxidative activity was not altered significantly upon Dexa treatment in ARP1 (**f**) and H929 (**g**) G6PD-OE cells. **h-i** DHEA effectively increased ROS content in ARP1 (**h**) and H929 (**i**) G6PD-OE cells treated with Dexa. *p < 0.05 was considered statistically significant

We further assessed the efficacy of DHEA in combination with Dexa in reversing G6PD-mediated Dexa resistance in MM. The MTT assays demonstrated that DHEA enhanced the sensitivity of cells to Dexa in MM.1S, ARP1, and H929 G6PD-OE cells (Fig. 7a–c). The protein expression levels of G6PD were significantly decreased following the combined treatment with DHEA and Dexa compared with Dexa treatment alone (Fig. 7d, e). Furthermore, the intracellular oxidative activity in G6PD-OE cells before and after Dexa treatment was measured; Dexa treatment did not increase ROS generation upon G6PD overexpression in MM cells (Fig. 7f, g). Intriguingly, the combination treatment of DHEA and Dexa significantly increased the ROS levels in the G6PD-OE cells (Fig. 7h, i), suggesting that DHEA treatment may reverse Dexa resistance induced by upregulated expression of G6PD.

## Discussion

The role of G6PD in preventing hemolysis of mammalian red blood cells (erythrocytes), which lack both a nucleus and mitochondria, is well-established. G6PD-deficient individuals are susceptible to the development of favism, neonatal jaundice and infection/drug-induced hemolysis primarily due to oxidative stress [30]. An increasing body of evidence in the last decade has documented the aberrantly high expression of G6PD in several types of cancers [11, 13, 15, 31]. Thus, our study was aimed to determine the oncogenic role of G6PD in promoting cellular proliferation, survival and chemotherapeutic drug resistance in MM.

We found that G6PD played a critical role in increasing the viability of MM cells as well as healthy cells primarily through the mediation of anti-oxidative processes. Untargeted metabolomics and GEO analyses suggested high expression of G6PD resulted in poor clinical outcomes during MM progression (Fig. 1). In contrast to a previous study, which reported that a modest increase in G6PD protected cells from oxidative damage and extended the lifespan in mice [31], our CDX mouse model, established using the G6PD-OE cells, exhibited an increased tumor burden (Fig. 2e–g), while the 5TMM3VT mice treated with a G6PD inhibitor, RRX-001, exhibited prolonged survival (Fig. 3e). This difference in results may be dependent on the spatiotemporal expression patterns and activity of G6PD in normal/diseased tissues or the local microenvironment. The contributions of G6PD to the proliferation and survival of cancer cells occur at several levels. As the first and rate-limiting enzyme in PPP signaling, G6PD facilitates the production of ribose-5-phosphate as well as cytoplasmic NADPH, both of which are required for the massive amounts of biosynthesis of essential nucleic acids and fatty acids needed during the accelerated replication and progression of cancer cells. Secondly, it is well-established that NADPH is an extremely important cellular antioxidant in the maintenance of redox homeostasis. As one class of the primary highly-bioactive and easily-diffusible small signaling molecules, including the superoxide anion O_2_^−^, hydrogen peroxide H_2_O_2_, and hydroxyl radical OH•, ROS play a dual role of either facilitating cancer cell proliferation, increasing metabolism and adaptation to hypoxia or triggering oxidative damage-induced cell death [32]. As shown in Fig. 5, the antioxidant capacity of G6PD-OE MM cells was increased to counteract hyperactive ROS generation during cellular transformation and tumorigenesis. Thirdly, the unique hallmark of cancer cells of relatively high turnover of ROS production and scavenging makes them more sensitive to alterations of ROS levels or the redox environment. This partially explains why MM cells are resistant to Dexa, which is a commonly used immunosuppressive anti-tumor drug able to enhance ROS-producing capacity and apoptosis of myeloid cells as well as osteoblasts [33, 34]. Consequently, knockdown or inhibition of G6PD improved the susceptibility of MM cells to Dexa and aggravated cell death (Fig. 3). Consistently, McBrayer et al. recently reported that elevated G6PD expression was necessary for the maintenance of resistance to nucleoside analogues (8-amino- and 8-chloro-adenosine) in multiple B-lineage lymphoid malignancies, and RNAi-mediated suppression of G6PD could sensitize resistance to 8-substituted adenosine analogues in MM cell lines [29]. Therefore, targeting G6PD may be a promising treatment for the management of MM. Okabe S et al. also reported that dual targeting of the PI3K/Akt pathway and histone deacetylase using fimepinostat (CUDC-907), may be a potential strategy for the treatment of MM [35].

From a broader perspective of metabolic reprogramming in cancer, the rewired metabolic network may enable cancer cells to evolve to utilize a variety of unconventional nutrient sources for survival and growth [36], in addition to the aerobic glycolysis (termed the Warburg Effect) in the cytoplasm [14]. Elsaadi S et al. found that human myeloma cells with phosphoglycerate dehydrogenase (PHGDH, the first and rate-limiting enzyme in the de novo serine synthesis pathway) expression knocked down exhibited increased sensitivity to BTZ and lower intracellular redox capacity [37]. Therefore, focusing on the dysregulation of metabolic pathways may be an important research strategy for improving the efficacy of MM therapeutics. Elevated G6PD levels allow cancer cells to hijack and reprogram the intracellular metabolic pathways, which influences DNA replication, cell division, redox equilibrium, and proliferation to obtain an advantageous condition, especially during chemical therapy. Since cancer cells generally exhibit high ROS levels or exposure, the rewired metabolic network of PPP may enable cancer cells to counteract persistent oxidative stress by NADPH reduction, as NADPH is the direct or indirect donor of several reductive metabolites including thioredoxins, glutathione, glutaredoxins, peroxiredoxins, and glutathione peroxidases [38]. Interestingly, a recent study using G6PD-knockdown cells demonstrated that G6PD neutralized oxidative stresses by supporting reductive glutamine metabolism and activating the AMPK pathway to balance the NADH/NAD^+^ ratio [39]. Therefore, the critical role of enzymatic G6PD in balancing cellular redox in cancer cells is supported by our results and those of other groups. We also provided evidence for the participation of the Wnt/β-catenin pathway in G6PD-induced proliferation and Dexa-resistance in MM cells in a non-enzymatic manner (Fig. 6 and Additional file 3: Fig. S4). This finding is consistent with the reports that oncogenic Wnt/β-catenin signaling promotes cancer cell growth, migration, and/or invasion in several solid and hematological cancers in vitro and in vivo [40, 41], despite an opposing relationship in the case of melanoma [42]. However, we could not exclude the involvement of other signaling pathways potentially involved in G6PD-induced MM cell growth and drug resistance, such as mTOR signaling (Additional file 2: Fig. S2). This is in agreement with a previous study on acute myeloid leukemia on the interplay between G6PD inhibition and mTORC1 activity [43], since mTOR is a conserved serine/threonine kinase controlling cell growth and metabolism in response to nutrients, growth factors, cellular energy, and stress [44]. In addition, G6PD transcription and activation of the PPP in turn can be mediated by Wnt/β-catenin signaling activation involving c-MYC, consequently conferring oxaliplatin chemoresistance in HCC cells [45]. There is an intricate interplay between Wnt/β-catenin and oxidative ROS [46], and Wnt/β-catenin signaling also plays key roles in metabolic reprogramming and drug resistance [47]. Thus, it is plausible to speculate that G6PD may well coordinate the enzymatic/non-enzymatic supply of necessary cellular energy and support anti-oxidant defense during tumorigenesis, which can be translated into MM therapy in the future.

Clinically, the inhibition of G6PD has been proven to be a useful strategy in reversing chemotherapy resistance, such as through the use of the competitive G6PD inhibitor 6-aminonicotinamide (6-AN) [48] and the uncompetitive G6PD inhibitor DHEA [19, 20]. The results from the untargeted metabolomics analysis and ELISA revealed that DHEAS levels were significantly decreased in the serum of the MM group compared with the Ctrl group (Fig. 1a–d). Furthermore, after G6PD-OE cells were treated with DHEA, G6PD protein expression and NADPH levels were significantly downregulated (Fig. 5d–g) while the ROS levels were significantly increased (Fig. 5i, k). As the active form of DHEAS, DHEA evidently reduced Dexa-induced oxidative stress in the renal cortex [49], possibly via inhibition of glucocorticoid receptor translocation into the nucleus [50, 51]. DHEA enhanced oxidative stress upon G6PD overexpression in MM cells and increased β-catenin expression (Fig. 7d–i). Although DHEA possesses anti-cancer, anti-obesity, and anti-inflammatory effects in animal models [52, 53], the clinical trials with DHEA were unsuccessful due to the need for high oral doses and the ready conversion of endogenous DHEA into the active androgens [54]. Additional studies are still required to investigate the relationship between G6PD, Wnt/β-catenin, and ROS-mediated drug resistance in MM.

## Conclusion

**Fig. 8:**
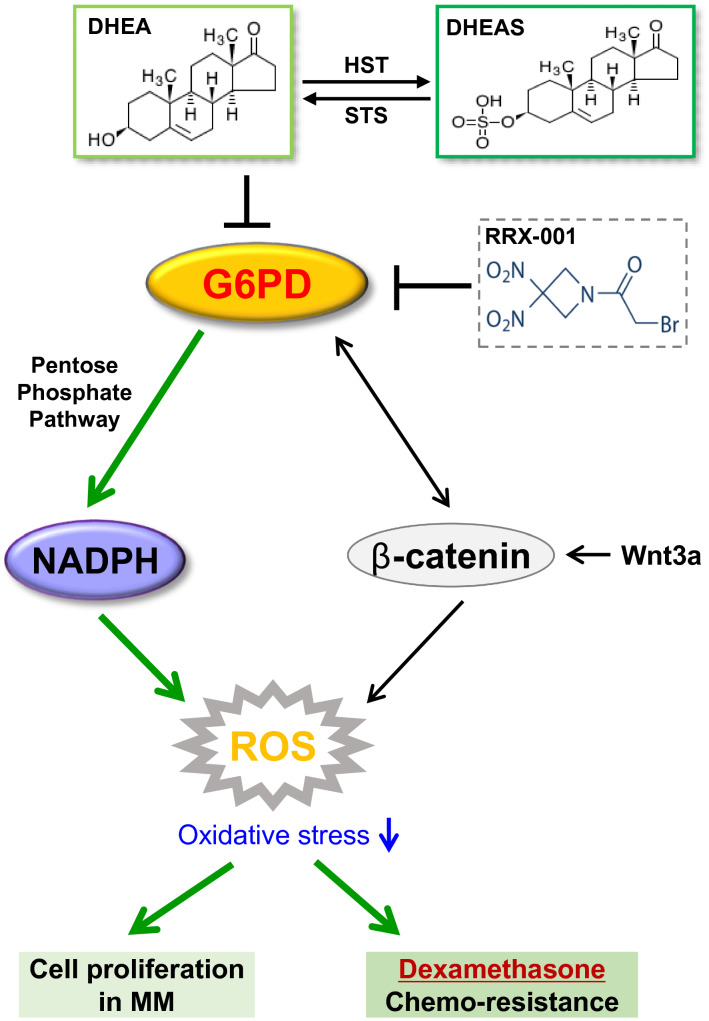
Schematic diagram of the potential mechanism of G6PD activity in MM

In conclusion, our findings demonstrated that upregulated expression of G6PD enhanced the intracellular anti-oxidative activity via increased generation of NADPH and reduced generation of ROS, which resulted in MM cells exhibiting antagonistic activity towards Dexa-induced apoptosis via modulation of the enzymatic PPP and non-enzymatic Wnt/β-catenin signaling pathway (Fig. 8). Targeting G6PD to regulate the cellular redox balance may provide a promising novel strategy for improving the management of MM patients and in the re-sensitization of relapsed MM patients to treatment.

## Supplementary Information

**Additional file 1: Table S1.** The clinical characteristics of 70 MM patients.

**Additional file 2: Fig S1.** Growth curves of ARP1/H929 G6PD-OE/KD cells cultured for 7 days. **Fig S2** WB analysis of mTOR and AKT in G6PD-OE/KD cells. **Fig. S3** Wnt3a partially restores the suppressed expression of G6PD and β-catenin by RRX-001.

**Additional file 3: Fig S4.** Grayscale analysis of WB in Fig. 6.

## Data Availability

Publicly available datasets were analyzed in this study. The data can be found here: https://www.ncbi.nlm.nih.gov/geo/query/acc.cgi?acc=GSE2658. The RNA-seq datasets generated for this study were also deposited in NCBI GEO database (accession number GSE172318). The raw data of untargeted metabolomics can be found here: https://www.ebi.ac.uk/metabolights/MTBLS5094.

## Abbreviations

G6PD: Glucose-6-phosphate dehydrogenase
BTZ: Bortezomib
CAR: Chimeric antigen receptor
CDX: Cell line-derived xenograft
Dexa: Dexamethasone
DHEAS: Dehydroepiandrosterone sulfate
DOX: Doxycycline
GEO: Gene expression omnibus
GO: Gene ontology
GR: Glucocorticoid receptor
GSH: Glutathione
HST: Hydroxysteroid sulfur transferase
IMiDs: Immunomodulatory drugs
MGUS: Monoclonal gammopathy of undetermined significance
MM: Multiple myeloma
NADPH: Nicotinamide adenine dinucleotide phosphate
NP: Normal bone marrow plasma cells
OPLS-DA: Orthogonal projections to latent structures discriminant analysis
OS: Overall survival
PIs: Proteasome inhibitors
PPP: Pentose phosphate pathway
ROS: Reactive oxygen species
STS: Steroid sulfatase
VIP: Variable importance in the projection
