# Supplementary material for: G6PD promotes cell proliferation and dexamethasone resistance in multiple myeloma via increasing anti-oxidant production and activating Wnt/β-catenin pathway
Source: Exp Hematol Oncol. 2022 Oct 21;11:77. doi: 10.1186/s40164-022-00326-6 (PMC9587560; doi:10.1186/s40164-022-00326-6)
Supplement: Supplementary file 1 — Additional file 1: Table S1. The clinical characteristics of 70 MM patients. [file 40164_2022_326_MOESM1_ESM.pdf]

Additional file 1: Table S1

Table S1 The clinical characteristics of 70 MM patients

**Characteristics**		**All evaluable patients, n = 70**
Age, years		62 (52-71)
Sex		
	Female	22
	Male	48
Therapies		
	Adriamycin	5 (7%)
	Bortezomib	49 (70%)
	Cyclophosphamide	52 (74%)
	Dexamethasone	69 (99%)
	Lxazomib	10 (14%)
	Melphalan	3 (4%)
	Thalidomide	18 (26%)
	Vincristine	38 (54%)
	Autologous stem-cell transplantation	24 (34%)
