# Supplementary material for: G6PD promotes cell proliferation and dexamethasone resistance in multiple myeloma via increasing anti-oxidant production and activating Wnt/β-catenin pathway
Source: Exp Hematol Oncol. 2022 Oct 21;11:77. doi: 10.1186/s40164-022-00326-6 (PMC9587560; doi:10.1186/s40164-022-00326-6)
Supplement: Supplementary file 2 — Additional file 2: Fig S1. Growth curves of ARP1/H929 G6PD-OE/KD cells cultured for 7 days. Fig S2 WB analysis of mTOR and AKT in G6PD-OE/KD cells. Fig. S3 Wnt3a partially restores the suppressed expression of G6PD and β-catenin by RRX-001. [file 40164_2022_326_MOESM2_ESM.pdf]

Additional file 2: Fig. S1-3

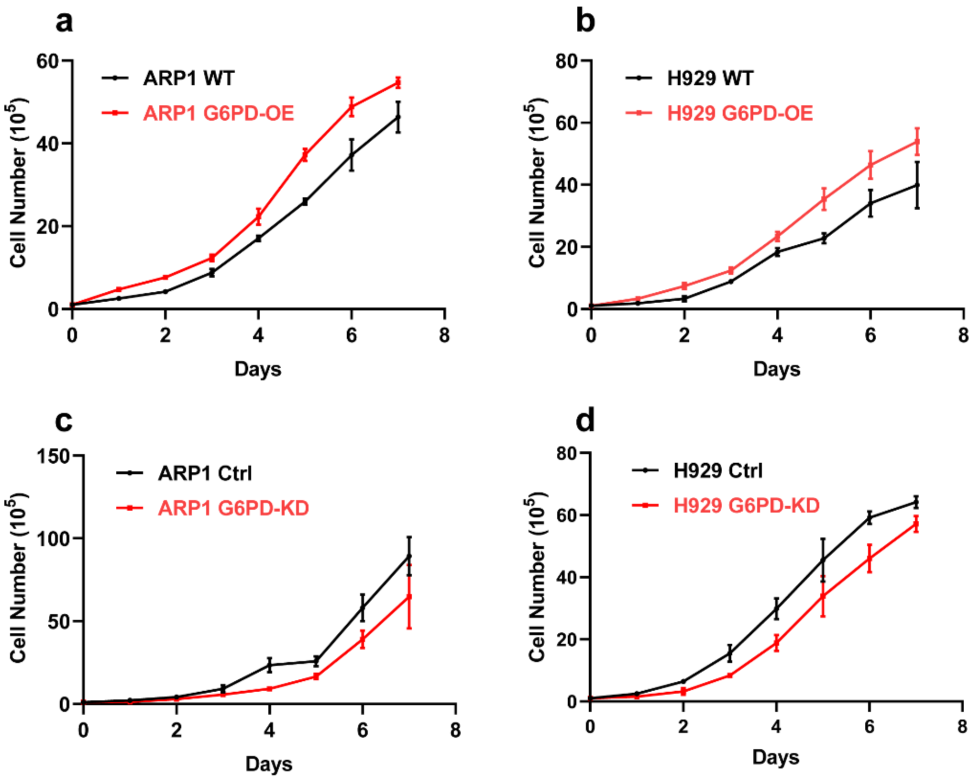

Fig. S1 Growth curves of ARP1/H929 G6PD-OE/KD cells cultured for 7 days.

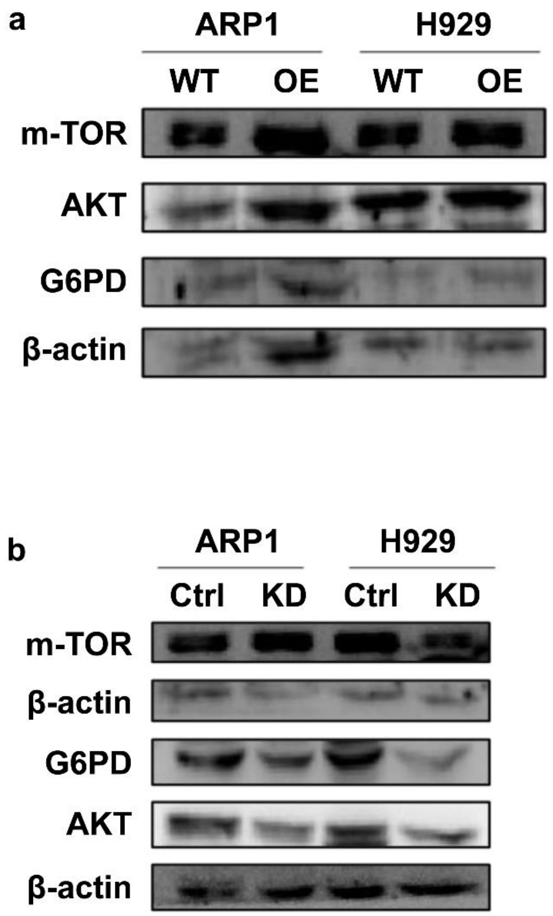

Fig. S2 WB analysis of mTOR and AKT in G6PD-OE/KD cells.

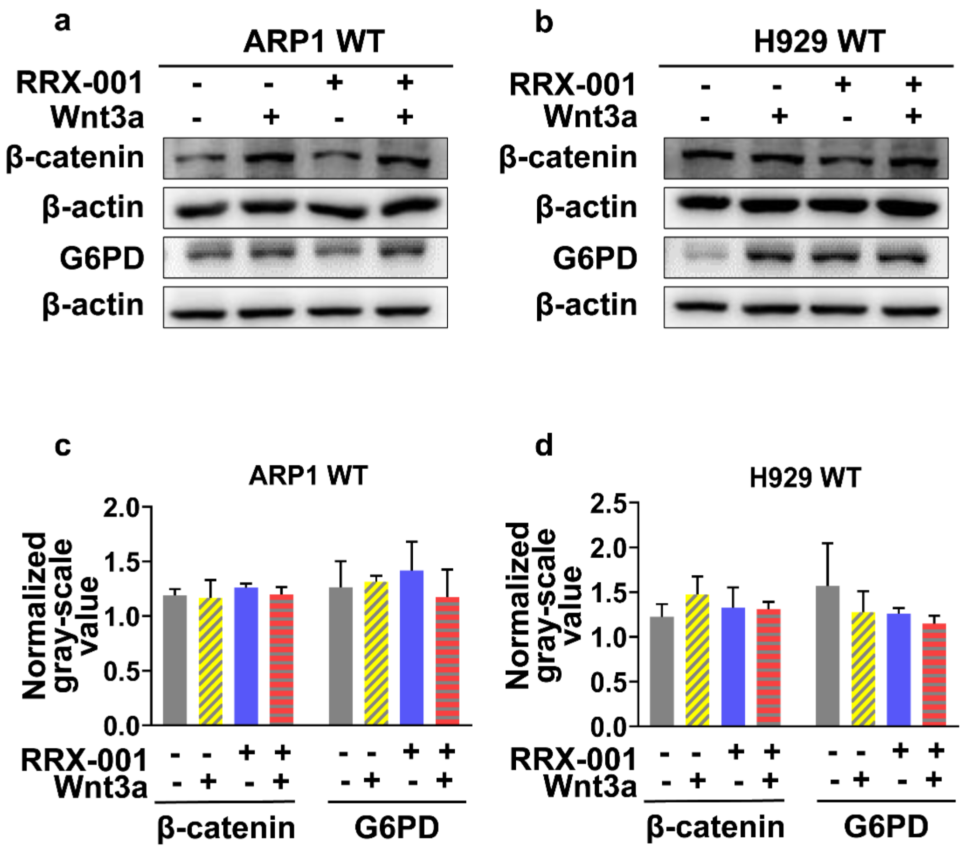

Fig. S3 Wnt3a partially restores the suppressed expression of G6PD and β-catenin by RRX-001. (a-b) Western blot showed the altered β-catenin and G6PD levels of MM cells in treatment with Wnt3a or/and RRX-001. **(c-d)** Quantitative gray scale analysis for (a) and (b).
