# Supplementary material for: G6PD promotes cell proliferation and dexamethasone resistance in multiple myeloma via increasing anti-oxidant production and activating Wnt/β-catenin pathway
Source: Exp Hematol Oncol. 2022 Oct 21;11:77. doi: 10.1186/s40164-022-00326-6 (PMC9587560; doi:10.1186/s40164-022-00326-6)
Supplement: Supplementary file 3 — Additional file 3: Fig S4. Grayscale analysis of WB in Fig. 6. [file 40164_2022_326_MOESM3_ESM.pdf]

Additional file 3: Fig. S4

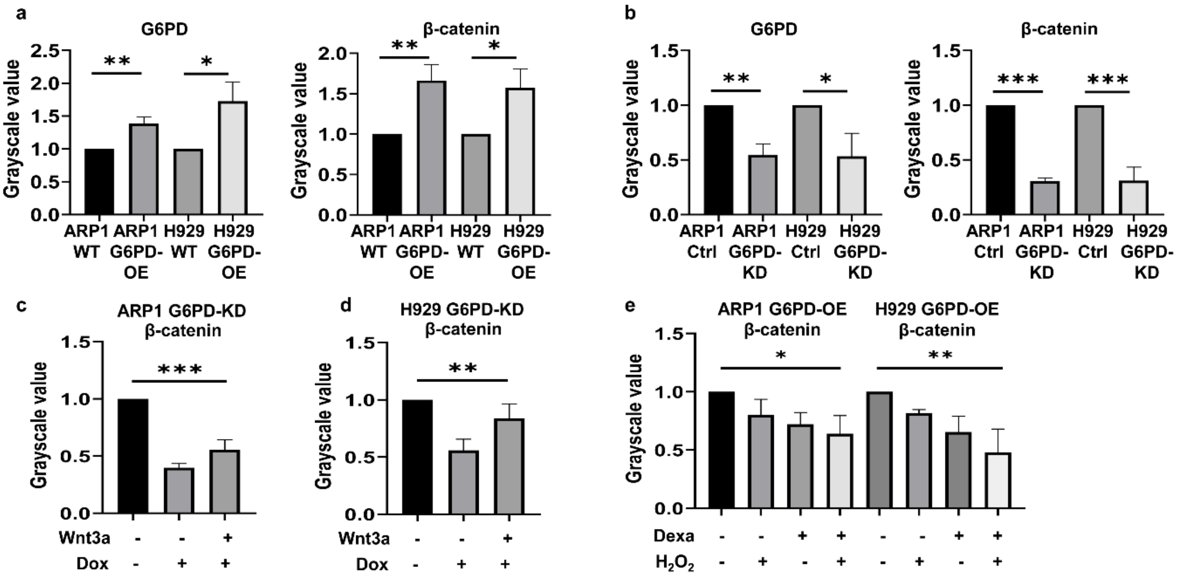

Fig. S4 Wnt/β-catenin signaling pathway is involved in G6PD-induced Dexa resistance in MM. (a-b) Grayscale analysis of Fig. 6a & 6b. The expression of G6PD was positively correlated with β-catenin in ARP1/H929 G6PD-OE (a) or G6PD-KD (b) MM cells. **(c-d)** Grayscale analysis of Fig. 6c & 6d. Wnt3a rescued the suppressed β-catenin expression in ARP1 (c) and H929 (d) G6PD-KD cells. **(e)** Grayscale analysis of Fig. 6e examined β-catenin levels in ARP1/H929 G6PD-OE cells treated with H_2O_2 and Dexa individually or in combination.
